# Just as they expected: How parents' expectations about their unborn child's characteristics provide a context for early transactions between parenting and child temperament

**DOI:** 10.3389/fpsyg.2022.942392

**Published:** 2022-09-20

**Authors:** Alithe L. Van den Akker, Mirjana Majdandzic, Wieke de Vente, Jessica J. Asscher, Susan Bögels

**Affiliations:** ^1^Research Institute Child Development and Education, University of Amsterdam, Amsterdam, Netherlands; ^2^Clinical Child, Family, and Education Studies, Utrecht University, Utrecht, Netherlands

**Keywords:** prenatal expectations, infant temperament, parenting, mothers, fathers, transactions

## Abstract

Prenatal expectations about what children will be like after birth may provide a context for how parents perceive their infant's actual temperament. We examined how these expectations and perceptions are associated and together predict early parenting behavior, with parenting behavior in turn predicting changes in temperament. Reports of 125 families (*N* = 122 fathers; *N* = 123 mothers; sample 1) about their expectations of their unborn child's temperament (negative affectivity, surgency, regulation, T1), their infant's temperament at 4 and 12 months post-partum (T2 and T3), and their hostile, responsive, warm, and overprotective parenting (T2) were included. We also included data from an independent sample of 168 mothers (sample 2), with the same measures, except that mothers reported on Big Five personality traits at T1. Results indicated that in both samples, parents' expectations were positively associated with perceptions of infant temperament. Prenatal expectations and newborn temperament independently predicted parenting behavior, and maternal and paternal parenting in turn predicted infant temperament at T3, controlling for infant temperament at T2. Although overall findings indicated associations between (expectations of) a more difficult temperament and more negative/less positive parenting, significant combinations of specific traits and parenting behaviors were sample-specific—indicating that more research is necessary to draw a conclusion about specific links. Both maternal and paternal expectations about their unborn child's temperament appear to carry over into the postpartum reality and provide a context for shaping early interactions between caregivers and their children, which may further shape the developing temperament of the child.

## Introduction

Parents' prenatal expectations provide an important context for early family life and may affect their parenting behavior and the newborn's unfolding temperament. While most pregnant women have positive expectations about their life with their newborn child, some women mostly worry that having a baby will negatively impact their life (Robakis et al., 2015). Pregnant women's expectations about their life after childbirth have previously been shown to predict their postpartum adjustment, with negative expectations associated with lower postpartum adjustment overall (Lawrence et al., 2007; Henshaw et al., 2014). Whereas previous studies have mostly investigated how prenatal expectations are associated with parental (mainly mothers') adjustment after childbirth, we examine how both mothers' and fathers' prenatal expectations of their children's characteristics are associated with their parenting behavior toward the newborn. Additionally, we examine how this early caregiving may have a lasting impact on the developing child, by predicting further development of the child's temperament traits across the first year of life.

### Prenatal expectations of child temperament and parenting behavior

Most studies on prenatal expectations have included general expectations about what it will be like to care for the baby (Kalmuss et al., 1992; Harwood et al., 2007; Henshaw et al., 2014; Robakis et al., 2015). Overall, the conclusion points to negative expectations carrying forward into the post-partum period, predicting depressed mood and lower marital relationship satisfaction after the child is born. A study that specifically investigated how expectations about the child's temperament traits were associated with post-partum adjustment found that when mothers expected their child to have a more difficult temperament overall, they reported a decline in marital satisfaction across the transition to parenthood (Lawrence et al., 2007). Expectations about child temperament may also be predictive of early parenting behavior toward the newborn, as many studies have supported the idea that after the child is born, child temperament traits elicit differences in parenting behavior (for a review, see Kiff et al., 2011).

Research of infant temperament has mostly converged on a model including three higher-order traits: Negative emotionality—indicating how easily children become distressed, fearful, and sad; Surgency—the tendency to experience positive emotions, have a high activity level and approach tendencies in social situations; and regulation—assessing attentional control and soothability (Gartstein and Rothbart, 2003). Children who are high on Negative emotionality and/or low on Regulation can be frustrating to deal with, and parents may exhibit hostile behavior toward children who are quick to cry (Scaramella et al., 2008) or not easily soothed (Morrell and Murray, 2003). At the same time, parents may display overprotective behavior in an effort to prevent their children from becoming upset (Booth-LaForce and Oxford, 2008). Additionally, parents may experience difficulty in establishing a positive relationship with their child and may report less warmth and responsivity (Mills-Koonce et al., 2007). Conversely, interacting with children who are high on Surgency might be rewarding for parents; when a child enjoys the interaction and exhibits positive emotions, parents may display more warmth in return. Parents may also indicate they are more responsive to the needs of their child, as they may interpret their child's behavior as positive feedback about their own parenting competencies.

In addition to actual infant temperament, parental expectations of infant temperament may be important in determining parenting behavior, because how parents experience their infants' temperament is likely to be partially determined by their own prenatal expectations about the infant's temperament. The same level of Regulation may, for instance, be interpreted differently by mothers when it is higher than they expected—a positive surprise—than when it is lower than expected—a negative surprise. Whether mothers are positively or negatively surprised may in turn impact how they treat their child. Parents who experience a positive surprise may exhibit more competent parenting, characterized by more warmth, responsiveness, less hostility, and overprotection. With regards to more general expectations about life after childbirth, a negative surprise has indeed been associated with maladjustment, with greater discrepancies between expectations and actual experiences, for instance, associated with a decline in relationship adjustment and an increase in depression postpartum (Kalmuss et al., 1992; Harwood et al., 2007).

Research into predictors of early caregiving is important, as early caregiving has enduring consequences for child development (Fraley and Roisman, 2015). Specifically, with regard to temperament development, evidence is accumulating that parenting behavior is not only shaped by child temperament traits, but also impacts the development of the child's temperament characteristics (e.g., Van Den Akker et al., 2010). Most evidence overall points to mutually reinforcing cycles of associations, with a more easy temperament predicting more positive parenting and this in turn predicting the development of a more easy temperament, and a similar transaction for negative parenting and a more difficult temperament. However, links between specific parenting behaviors and child traits are not always replicated (Kiff et al., 2011).

### This study

The overall aim of this study was to examine how prenatal expectations of child characteristics play a role in the early establishment of transactional associations between temperament and parenting. We used data from two longitudinal studies (Project 1: *N* = 122 fathers/123 mothers/Project 2: *N* = 168 mothers) that both included a prenatal assessment of expected child temperament characteristics (T1), and actual child characteristics several months post-partum (T2 at 4 months for Project 1; at 6 months for Project 2), and when infants were 12 months old (T3). We sought to answer the following research questions: What levels of temperament traits do expecting parents expect in their future child, and are expected trait levels associated with actual traits of their newborns? Do infant temperament traits interact with prenatal expectations about child traits to predict early parenting behavior, with this parenting behavior in turn predicting infant temperament at 12 months? Data from Project 1 were used to study these questions in fathers and mothers. Data from Project 2 were used for a conceptual replication in mothers with the same hypotheses, but with different, conceptually related, expected characteristics assessed (Tackett et al., 2013). We replaced expected Negative Affectivity with expected Neuroticism, expected Regulation with expected Conscientiousness, and expected Surgency with expected Extraversion.

**Figure 1:**
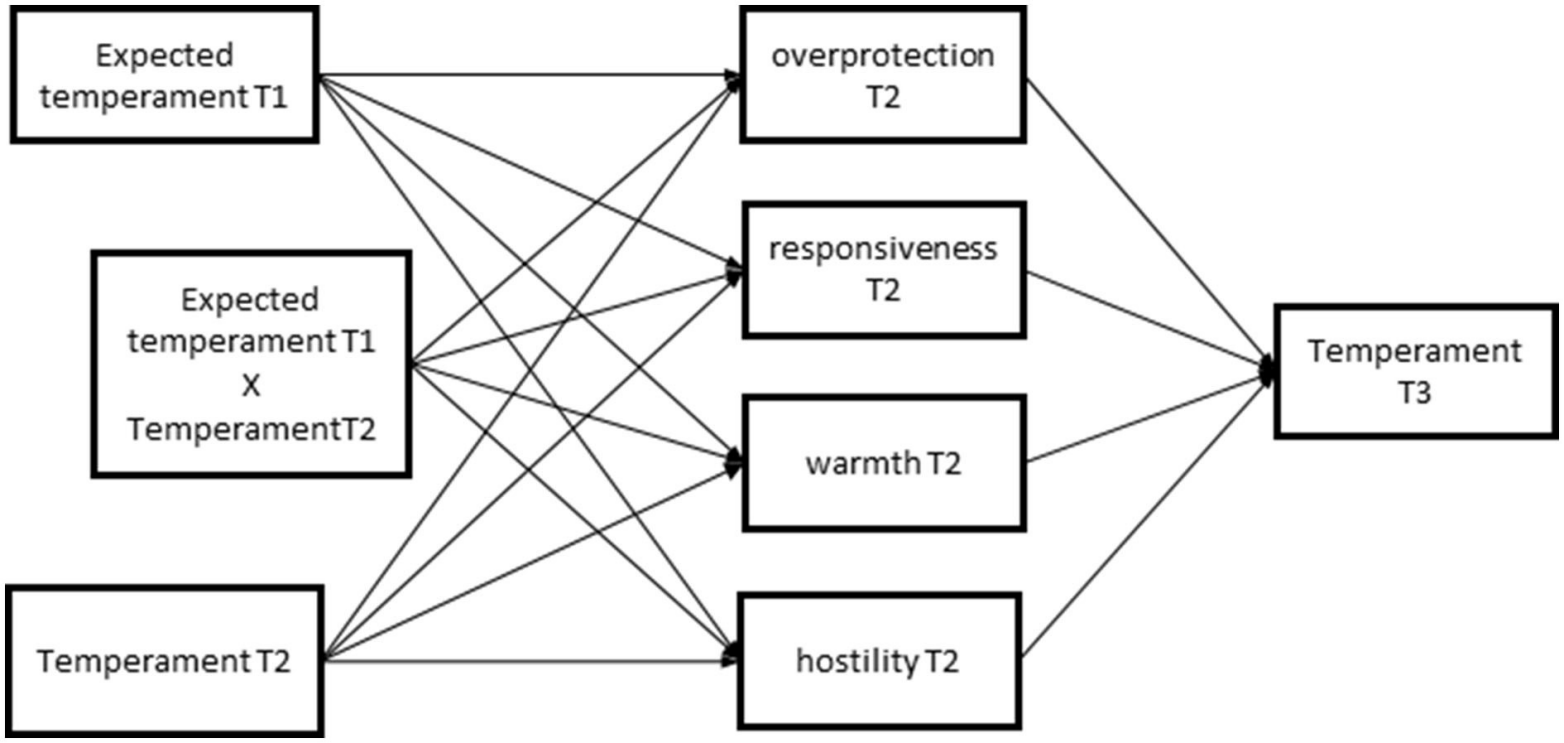
Graphical representation of the estimated models including the interaction. Please note a direct effect from temperament T2 to temperament T3 was also included, as were squared terms for expected temperament and temperament at T2, and covariances between the parenting variables – but are not depicted in this figure for the sake of clarity. In our initial plan we had included covariances between expected temperament and temperament T2 and their interaction, but these resulted in estimation problems and had to be removed.

We formulated the following hypotheses: First, in line with previous findings of relatively positive prenatal expectations overall (Robakis et al., 2015), parents will expect their future child to have relatively favorable characteristics, with mean expected levels of Regulation and Surgency above the midpoint of the scale and levels of Negative Affectivity below the midpoint of the scale (for the Big Five traits from Project 2, we also included Agreeableness, and Openness, with expected values above the midpoint); Second, expecting parents' expectations about infant temperament will be positively associated with reported temperament of the newborn, as parents may use knowledge of their own (and/or their partners') characteristics to base their expectations on. Some evidence from previous studies also indicates that parents' expectations of infant temperament are associated with temperament assessed after the child is born (Mebert and Kalinowski, 1986; Zeanah et al., 1986; Diener et al., 1995). Third, infants' temperament at 4 months is associated with parenting behavior at 4 months, with (a) higher levels of infant Negative Affectivity associated with more hostility and overprotection, and less warmth and responsivity, (b) higher levels of Surgency associated with less overprotection and more warmth and responsivity, and (c) lower levels of Regulation associated with more hostility, and less warmth and responsivity. Fourth, parents' prenatal expectations about temperament and infant temperament assessed at 4 months (6 in Project 2) interact to predict parenting behavior as follows: When infants' temperament is easier to deal with than parents expect (i.e., lower Negative Affectivity, higher Regulation, or higher Surgency)—a positive surprise, they report more warmth and responsivity. When infants' temperament is more difficult to deal with than parents expect (i.e., higher Negative Affectivity, lower Regulation, or lower Surgency)—a negative surprise—they report more hostility, less warmth and responsivity, and more overprotection. Fifth, parenting at 4–6 months in turn predicts development in infant temperament from 4/6 months to 12 months. Parents' higher levels of hostility and overprotection predict lower levels of infant Regulation, higher levels of Negative Affectivity, and lower levels of Surgency, controlling for previous levels of the same temperament dimensions. Parents' higher levels of warmth and responsivity predict lower levels of infant Negative Affectivity and higher levels of Surgency and Regulation, controlling for previous levels of the same temperament dimensions. Sixth, we assessed mediation and expected that temperament expectations at T1, infant temperament at T2, and the interaction of these temperament characteristics would be associated with temperament at T3 *via* the parenting variables at T2. For a conceptual model, see Figure 1.

## Materials and methods

### Sample

In Project 1, the first three waves from the longitudinal study of The Social Development of Children (Majdandžić et al., 2016) were included. Couples who were expecting their first child were recruited through leaflets provided by midwives in Amsterdam and in cities within a range of 50 km around it, at pregnancy courses, at baby shops, and through advertisements in magazines and on websites on parenthood. Recruitment was done by a team of researchers and research assistants and took place from June 2007 to June 2009 (T1). There were follow-up data waves at the child's age of 4 months (T2), 1 year (T3), 2.5 years (T4), 4.5 years (T5), and 7.5 years (T6). At the data waves, families participated with their children in lab tasks and home visits and filled out questionnaires on paper. After completing a data wave, families received a 20 Euro gift voucher, and (at the postnatal data waves) a small present for the child and a recording of the laboratory sessions. Of the 151 couples for whom either the father or the mother provided information for the expected child characteristics at the prenatal assessment (T1), we included as part of the longitudinal sample those families for whom either the father or the mother also participated at T2, resulting in a total sample size of *N* = 125 families (*n* = 122 fathers; *n* = 123 mothers; babies: 69 girls (55%) 56 boys). Of these, *n* = 114 families also participated at T3 (*n* = 110 fathers; *n* = 113 mothers). The vast majority of parents were of Dutch origin (90% of mothers and 95% of fathers). Educational level was fairly high; 20% of mothers and 38% of fathers had finished vocational training, and 63% of mothers and 62% of fathers had an associate degree or higher. Mothers' mean age at Time 2 was 32.08 years, *SD* = 4.10, and fathers' mean age was 34.97 years, *SD* = 5.32.

In Project 2, mothers who were expecting a baby were recruited from December 2013 through April 2014 (T1). There were follow-up data waves at 6 months (T2), 1 year (T3), and 3.5 years (T4), and we used the data of the first three waves here. Students collected data as part of a research practicum, and recruited participants online, through websites for expecting women and young parents, Facebook, and face-to-face in Amsterdam. Mothers who participated in wave 1 were eligible to win a 100 Euro gift certificate, and for each subsequent wave, mothers could win a 50 Euro gift certificate. Mothers filled out an online questionnaire (Qualtrics). Of the 560 participants who participated at T1, we included those who also participated at T2, resulting in a final sample size of *N* = 168. Of these, *n* = 130 also participated at T3. Of the final sample, 2% were single mothers. Educational levels were as follows: 30% had finished vocational training and 70% had an associate degree or higher. Most mothers were of Dutch origin (96%).

### Measures

For an overview of the measures included in this study, see Table 1.

**Table 1:** Overview of included measures.

	**T1**			**T2**			**T3**		
	**During pregnancy**			**4 months** **post-partum**		**6 months post-partum**	**12 months** **post-partum**		
	**S1 mothers**	**S1 fathers**	**S2 mothers**	**S1 mothers**	**S1 fathers**	**S2 mothers**	**S1 mothers**	**S1 fathers**	**S2 mothers**
Regulation	X	X		X(c)		X	X	X	X
Surgency	X	X		X(c)		X	X	X	X
Negative affectivity	X	X		X(c)		X	X	X	X
Big five personality			X						
Hostility				–	X	X			
Overprotection				X	X	X			
Responsivity				X	X	X			
Warmth				X	X	X			

S1, sample 1; S2, sample 2. (c) Indicates measures were combined. Dashes indicate the measure was not included because it was not sufficiently reliable.

### Expected child traits

In Project 1, parents' expectations about their child's temperament were assessed by having mothers and fathers fill out a balanced set of representative items selected from the following instruments: the Infant Behavior Questionnaire-Revised (Gartstein and Rothbart, 2003), the Early Childhood Behavior Questionnaire (Putnam et al., 2006), and the Children's Behavior Questionnaire (Rothbart et al., 2001) at Time 1. We computed higher-order scales for expected infant Negative Affectivity (13 items, example item: “I expect that my child has temper tantrums when s/he doesn't get what s/he wants”; discomfort: 3 items, sadness: 3 items, fear: 4 items, anger: 3 items), Regulation (15 items, example item: “I expect that my child can wait patiently when asked to wait for a desirable item”; inhibitory control: 3 items, attentional focusing: 3 items, attentional shifting: 3 items, low-intensity pleasure: 3 items, soothability: 3 items), and Surgency (19 items, example item: “I expect that my child gets very excited when given a new toy,” impulsivity: 3 items, shyness (recoded): 4 items, activity level: 3 items, approach: 3 items, high-intensity pleasure: 3 items). Items were rated on Likert-type scales ranging from 1 (*never*) to 7 (*always*). Cronbach's alphas for mothers' reports were 0.73 for expected Regulation, 0.70 for expected Negative Affectivity, and 0.85 for expected Surgency. For fathers' reports, Cronbach's alphas were 0.62 for expected Regulation, 0.73 for expected Negative Affectivity, and 0.81 for expected Surgency. Confirmatory factor analysis was performed on the lower-order scales for the expected temperament measures in JASP version 0.16 (Team, 2021). Results indicated that a three-factor solution, with residual covariances added when they were indicated by the modification indices and did not result in problems in estimating the model, provided a sufficient fit to the data for both mothers: χ^2^(70) = 112.93, CFI = 0.913, RMSEA = 0.069 [0.044, 0.092], and fathers: χ^2^(67) = 111.21, CFI = 0.909, RMSEA = 0.071 [0.047, 0.094]. All scales loaded significantly and in the expected direction, except for discomfort in the mother data, for which the loading was not significant (albeit in the expected direction). We decided not to remove these items from the scales to allow for comparability of the measures across mothers and fathers.

In Project 2, pregnant mothers filled out the Dutch version of the Ten Item Personality Inventory (TIPI) (Hofmans et al., 2008) about their child, which includes two items for each of the Big Five scales (Extraversion, example item: “extraverted, enthusiastic,” Agreeableness, example item: “critical, argumentative,” Conscientiousness, example item: “thorough, disciplined,” Neuroticism, example item: “fearful, easily upset,” Openness-to-experience, example item: “open to new experiences, active imagination”) (Gosling et al., 2003). Items were rated on Likert-type scales ranging from 1 (*not at all*) to 7 (*very much*). This version has been shown to be a valid alternative covering the five dimensions when time is limited (Hofmans et al., 2008). As this measure was designed to have the most coverage of the personality dimensions with the fewest items, this necessarily results in lower internal consistency than choosing items that measure the same aspect of the dimension (Hofmans et al., 2008). Consequently, alphas ranged from 0.22 for Conscientiousness to 0.46 for Neuroticism in the present sample. As models with <3 indicators per factor are subject to estimation problems (Kline, 2005, p. 114), confirmatory factor analysis of the TIPI was not attempted in this sample.

### Infant temperament

In Project 1, mothers and fathers filled out the Infant Behavior Questionnaire Revised (Gartstein and Rothbart, 2003) for children aged 4 months and 1 year. We computed higher-order scales for Negative Affectivity (59 items, example item: “When tired, how often did your baby show distress?”, sadness: 14 items, fear: 16 items, falling reactivity (reversed): 13 items, distress to limitation: 16 items), Regulation (60 items, example item: “How often during the last week did the baby stare at a mobile, crib bumper or picture for 5 min or longer?”, cuddliness: 17 items, duration of orienting: 12 items, low-intensity pleasure: 13 items, soothability: 18 items), and Surgency (60 items, example item:” When tossed around playfully how often did the baby laugh?”, activity level: 15 items, smiling and laughter: 10 items, vocal reactivity: 12 items, approach: 12 items, high-intensity pleasure: 11 items). Items were rated on Likert-type scales ranging from 1 (*never*) to 7 (*always*), with an option for when the item was not applicable. Cronbach's alphas were good, ranging from 0.80 to 0.91 for fathers, and from 0.85 to 0.89 for mothers. Mother and father reports were significantly correlated, range *r* = 0.25–0.64. We combined mother and father reports by averaging them to obtain robust measures of infant temperament at 4 months and 1 year.

In Project 2, mothers filled out the Infant Behavior Questionnaire Revised—Short form (Putnam et al., 2014) at child aged 6 months and 1 year. We computed higher-order scales for Negative Affectivity (25 items, example item: “When tired, how often did your baby show distress?”, sadness: 6 items, fear: 6 items, falling reactivity (reversed): 6 items, distress to limitation: 7 items), Regulation (26 items, example item: “How often during the last week did the baby stare at a mobile, crib bumper or picture for 5 min or longer?”, cuddliness: 6 items, duration of orienting: 6 items, low intensity pleasure: 7 items, soothability: 7 items), and Surgency (34 items, example item:” When tossed around playfully how often did the baby laugh?”, activity level: 7 items, smiling and laughter: 7 items, vocal reactivity: 7 items, approach: 6 items, high intensity pleasure: 7 items). Items were rated on Likert-type scales ranging from 1 (*never*) to 7 (*always*), with an option for when the item was not applicable. Cronbach's alphas were good, ranging from 0.81 to 0.91.

### Parenting behavior

In Project 1, mothers and fathers filled out the Comprehensive Parenting Behavior Questionnaire at 4 months (Majdandžić et al., 2008), and in Project 2, mothers filled out the same questionnaire at 6 months. We computed mean scale scores for hostility (6 items, example item: “When my child cries for a long time, I yell at him/her”), overprotection (9 items, example item: “I try to minimize sound around my child as much as possible”), responsivity (5 items, example item: “When my child cries, I know what's wrong”), and warmth (6 items, example item: “I regularly cuddle with my child”). Parents rated how much the items applied to them on Likert-type scales ranging from 1 (*not at all*) to 7 (*completely*). Except for hostility as reported by mothers in Project 1 (Cronbach's alpha = 0.46), Cronbach's alphas were acceptable to good, ranging from 0.64 to 77 for mother reports and 0.65 to 0.80 for father reports in Project 1, and from 0.61 to 71 for the mothers in Project 2. As we could not obtain sufficient reliability (i.e., >0.60) for maternal hostility in Project 1 by removing items, we excluded maternal ratings of this measure in Project 1 from further analysis.

### Analysis plan

We tested our first hypothesis—that parents have relatively favorable expectations of their future child's temperament—by testing (one-sided) whether the observed sample mean of each expected temperament scale differs significantly from the value representing the midpoint of the scale. To test Hypotheses 2–4, we fit three structural equation models in Mplus (Muthén and Muthén, 1998)—one for each expected temperament characteristic—according to the model as shown in Figure 1. For Project 1, separate models were fitted for mothers and fathers. Covariances between expected temperament at T1 and temperament at T2 were included (hypothesis 2), as well as covariances between the parenting variables at T2. The main effects of newborn temperament on parenting were tested (hypothesis 3). To study the effects of a positive or negative surprise (i.e., newborn temperament is less or more difficult than expected, respectively), we examined whether associations between newborn temperament and parenting were moderated by expected temperament (hypotheses 4 and 5). We therefore included the main effects of both expected and newborn temperament, quadratic terms for these main effects, and interaction effects between the expected temperament characteristics and newborn temperament at T2 (Laird and De Los Reyes, 2013). This approach has been shown to be preferable over for instance including different scores (Edwards, 2001). The main effects were mean-centered and quadratic and interaction terms were computed using mean-centered variables. To test mediation, indirect effects from expected temperament at T1 and infant temperament at T2, as well as their interaction, on temperament at T3, *via* parenting T2 were tested (hypothesis 6). The direct effects from Temperament T2 to Temperament T3 were controlled for.

To determine absolute model fit, we used the Root Mean Square Error of Approximation (RMSEA), with RMSEA <0.05 was considered a good fit and 0.05–0.08 as an acceptable fit (Browne and Cudeck, 1992), and the Comparative Fit Index (CFI), with CFI >0.95 is considered good fit (Hu and Bentler, 1999). Given that some of the variables were expected to be non-normally distributed (hostility and overprotection) and in view of missing data, we used Full Information Maximum Likelihood estimation with a Robust estimator. Outliers—outside the 1.5 Interquartile range—were winsorized to the nearest value within that range. The hypotheses and analysis plan were preregistered on the Open Science Framework (https://osf.io/k53zt/?view_only=221fbc060e5d4a11bc60ddf8f8a95d0e).

## Results

Descriptives of the study variables are presented in Table 2.

**Table 2:** Descriptives of the study variables.

	**Project 1**		**Project 2**
	**Mothers**	**Fathers**	**Mothers**
**Measure**	***M (SD)***	***M (SD)***	***M (SD)***
Exp. Negative affectivity/Neuroticism	3.32 (0.57)	3.28 (0.59)	2.71 (0.92)
Exp. Regulation/Conscientiousness	4.78 (0.51)	4.77 (0.44)	4.95 (0.91)
Exp. Surgency/Extraversion	4.94 (0.56)	4.90 (0.55)	5.19 (0.95)
Exp. Agreeableness	–	–	5.57 (0.84)
Exp. Openness-to-Experience	–	–	5.41 (0.88)
Overprotection T2	2.07 (0.55)	2.02 (0.57)	2.03 (0.55)
Responsiveness T2	4.18 (0.39)	3.83 (0.45)	4.56 (0.38)
Hostility T2	–	1.40 (0.39)	1.44 (0.40)
Warmth T2	4.80 (0.26)	4.61 (0.44)	4.97 (0.11)
	Composite		
Infant Negative affectivity T2	2.93 (0.57)		2.50 (0.75)
Infant Regulation T2	5.07 (0.37)		5.58 (0.56)
Infant Surgency T2	3.96 (0.50)		4.81 (0.72)
Infant Negative affectivity T3	2.98 (0.56)		2.72 (0.86)
Infant Regulation T3	4.86 (0.38)		5.38 (0.57)
Infant Surgency T3	4.68 (0.42)		5.16 (0.53)

Parenting dimensions were rated on scales ranging from 1 to 5. Temperament and personality scores were rated on scales ranging from 1 to 7.

### Expectations about temperament

**Figure 2:**
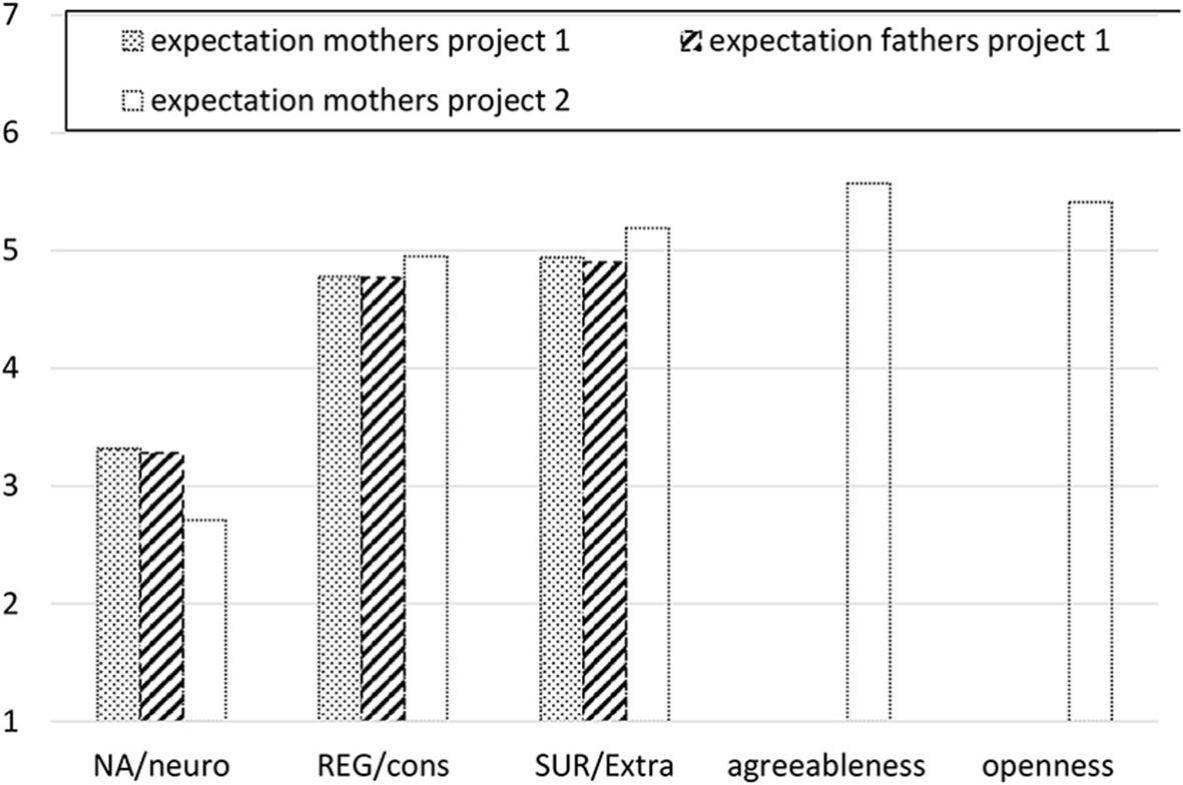
Expected temperament and personality traits scores. NA, Negative affectivity; REG, Regulation; SUR, Surgency.

As expected, both mothers and fathers in Project 1 had positive expectations about the child's temperament traits, with all means significantly different from 4, at *p* < 0.001. Both mothers and fathers expected their child to be lower on Negative Affectivity and higher on Surgency and Regulation than the midpoint of the scale. Paired samples *t*-tests indicated that mothers' and fathers' expectations did not differ significantly [*t*_Negative Affectivity_(113) = −0.15, *p* =0.879; *t*_Regulation_(113) = 0.43, *p* = 0.668; *t*_Surgency_(113) = 0.90, *p* = 0.369]. Results from Project 2 also confirmed our hypothesis, with all means significantly different from 4, at *p* < 0.001. Mothers expected their children to be more extraverted, agreeable, conscientious, and open to experience than the mid-point of the scale, and less neurotic. For a visual representation of expected levels of the traits see Figure 2.

### Expected temperament, parenting, and actual temperament

We fitted models with the main effects of expected temperament and newborn temperament as well as their squared terms and interactions, on parenting, with parenting in turn predicting 12-month temperament (Figure 1). For model fit statistics, see Table 3. In these models, we first examined associations between expected and actual temperament (hypothesis 2). We predicted that expecting parents' expectations about infant temperament would be positively associated with the reported temperament of the newborn. In both Project 1 and Project 2, we found that higher levels of expected Regulation were associated with higher levels of actual Regulation of the newborn (see Table 4). In Project 2, mothers' higher expected Surgency was also associated with higher Surgency at 4–6 months, and in Project 1, fathers' expected Negative Affectivity was associated with higher actual Negative Affectivity. For estimates of the associations, see Table 4.

**Table 3:** Model fit statistics.

	**Project 1**							**Project 2**		
	**Mothers**			**Fathers**				**Mothers**		
**Model**	**RMSEA**	**CFI**	**χ^2^ (*df*)**	**RMSEA**	**CFI**	**χ^2^ (*df*)**		**RMSEA**	**CFI**	**χ^2^ (*df*)**
Negative affectivity	0.069	0.909	15.56 (10)	0.000	1.000	8.13 (10)	Neuroticism	0.058	0.971	10.91 (7)^a^
Regulation	0.000	1.000	6.64 (10)	0.000	1.000	4.83 (10)	Conscientiousness	0.000	1.000	4.98 (10)
Surgency	0.060	0.933	14.20 (10)	0.000	1.000	8.57 (10)	Extraversion	0.068	0.925	17.87 (10)

^a^The squared term for the main effect of infant temperament at T2 was removed to be able to fit the model.

**Table 4:** Associations between expected temperament T1 and infant temperament T2.

	**Project 1**				**Project 2**	
	**Mothers**		**Fathers**		**Mothers**	
**covariances**	**σ (*SE*)**	***p***	**σ (*SE*)**	***p***	**σ (*SE*)**	***p***
Exp. NA/neuro← → NA T2	0.01 (0.09)	0.910	0.24 (0.07)	0.001	0.09 (0.08)	0.239
Exp. REG/cons ← → REG T2	0.25 (0.10)	0.013	0.15 (0.08)	0.084	0.18 (0.07)	0.015
Exp. SUR/extra ← → SUR T2	0.14 (0.09)	0.118	0.13 (0.08)	0.093	0.20 (0.07)	0.006

NA, Negative Affectivity; REG, Regulation; SUR, Surgency; neuro, Neuroticism; cons, Conscientiousness; extra, Extraversion.

Our third hypothesis addressed the associations between infant temperament at T2 and parenting at T2. In sample 1, we did not find any significant associations between infant Negative Affectivity at 4 months and parenting, whereas in sample 2 we found two associations in the expected direction: higher infant Negative Affectivity at 6 months was associated with lower responsivity and higher hostility (see Table 5). Of note, we could not include hostility for mothers in Sample 1, as this measure was not sufficiently reliable.

**Table 5:** Effects of expected temperament T1, infant temperament T2, and their interaction on parenting.

	**Overprotection T2**		**Responsivity T2**		**Hostility T2**		**Warmth T2**	
**Project 1**	**β (S.E.)**	***p***	**β (S.E.)**	***p***	**β (S.E.)**	***p***	**β (S.E.)**	***p***
**Mother**								
**Negative Affectivity**								
Exp. NA	0.17 (0.09)	0.052	−0.10 (0.10)	0.343	–	–	−0.22 (0.11)	0.046
NA T2	0.10 (0.09)	0.303	−0.17 (0.10)	0.090	–	–	0.15 (0.09)	0.097
[Exp. NA]^2^	−0.02 (0.08)	0.859	−0.06 (0.09)	0.516	–	–	−0.12 (0.12)	0.349
[NA T2]^2^	−0.08 (0.10)	0.389	0.09 (0.08)	0.268	–	–	−0.04 (0.11)	0.730
Exp. NA × NA T2	−0.01 (0.08)	0.877	0.13 (0.11)	0.234	–	–	0.08 (0.12)	0.518
**Regulation**								
Exp. REG	−0.17 (0.09)	0.048	0.31 (0.07)	<0.001	–	–	0.18 (0.10)	0.074
REG T2	−0.06 (0.10)	0.558	0.31 (0.06)	<0.001	–	–	0.00 (0.11)	0.997
[Exp. REG]^2^	0.09 (0.11)	0.393	−0.03 (0.08)	0.708	–	–	0.13 (0.09)	0.142
[REG T2]^2^	0.16 (0.10)	0.115	0.30 (0.08)	<0.001	–	–	0.13 (0.12)	0.272
Exp. REG × REG T2	0.17 (0.11)	0.112	−0.27 (0.08)	0.001	–	–	−0.10 (0.15)	0.510
**Surgency**								
Exp. SUR	−0.19 (0.09)	0.046	0.24 (0.09)	0.004	–	–	0.01 (0.12)	0.950
SUR T2	−0.07 (0.09)	0.436	0.16 (0.10)	0.107	–	–	0.29 (0.10)	0.002
[Exp. SUR]^2^	−0.07 (0.11)	0.517	−0.07 (0.07)	0.301	–	–	−0.40 (0.09)	<0.001
[SUR T2]^2^	−0.02 (0.09)	0.796	0.02 (0.11)	0.839	–	–	−0.11 (0.10)	0.248
Exp. SUR × SUR T2	0.11 (0.10)	0.306	−0.02 (0.09)	0.829	–	–	0.03 (0.11)	0.806
**Father**								
**Negative affectivity**								
Exp. NA	0.24 (0.08)	0.004	−0.28 (0.09)	0.002	0.23 (0.10)	0.017	−0.17 (0.12)	0.138
NA T2	0.05 (0.11)	0.649	0.16 (0.10)	0.125	0.14 (0.08)	0.084	−0.01 (0.11)	0.930
[Exp. NA]^2^	0.11 (0.09)	0.224	0.06 (0.10)	0.554	−0.02 (0.09)	0.823	−0.08 (0.12)	0.483
[NA T2]^2^	0.00 (0.13)	0.978	0.18 (0.10)	0.074	−0.14 (0.09)	0.136	−0.01 (0.09)	0.936
Exp. NA × NA T2	−0.08 (0.11)	0.436	−0.12 (0.10)	0.257	0.13 (0.09)	0.181	0.14 (0.13)	0.267
**Regulation**								
Exp. REG	−0.06 (0.08)	0.446	0.29 (0.10)	0.003	−0.04 (0.09)	0.630	0.17 (0.11)	0.136
REG T2	−0.03 (0.10)	0.733	0.18 (0.11)	0.101	−0.13 (0.09)	0.172	0.19 (0.07)	0.011
[Exp. REG]^2^	0.03 (0.08)	0.746	−0.02 (0.07)	0.775	0.02 (0.09)	0.843	−0.07 (0.08)	0.383
[REG T2]^2^	0.02 (0.10)	0.863	−0.05 (0.11)	0.661	−0.07 (0.10)	0.510	0.06 (0.07)	0.370
Exp. REG × REG T2	−0.04 (0.10)	0.680	0.11 (0.13)	0.408	0.06 (0.09)	0.475	−0.08 (0.12)	0.499
**Surgency**								
Exp. SUR	−0.19 (0.10)	0.073	−0.01 (0.10)	0.932	−0.03 (0.10)	0.739	0.16 (0.10)	0.109
SUR T2	0.09 (0.08)	0.253	0.30 (0.10)	0.002	0.03 (0.09)	0.771	0.11 (0.14)	0.412
[Exp. SUR]^2^	−0.11 (0.12)	0.339	0.06 (0.10)	0.538	0.02 (0.10)	0.816	0.07 (0.09)	0.434
[SUR T2]^2^	−0.06 (0.08)	0.479	−0.02 (0.10)	0.834	−0.03 (0.08)	0.747	−0.13 (0.16)	0.425
Exp. SUR × SUR T2	−0.03 (0.09)	0.730	−0.06 (0.09)	0.493	0.16 (0.09)	0.059	0.01 (0.09)	0.888
**Project 2**								
**Neuroticism/**								
Exp. Neuro.	0.10 (0.07)	0.150	−0.11 (0.07)	0.152	0.02 (0.07)	0.777	0.02 (0.13)	0.897
NA T2	0.04 (0.08)	0.592	−0.18 (0.08)	0.027	0.38 (0.08)	<0.001	0.01 (0.05)	0.809
[Exp. Neuro]^2^	−0.13 (0.07)	0.072	−0.16 (0.08)	0.038	0.07 (0.08)	0.388	−0.07 (0.13)	0.590
[NA T2]^2^	–	–	–	–	–	–	–	–
Exp. Neuro. × NA T2	0.04 (0.08)	0.650	0.10 (0.07)	0.163	−0.03 (0.09)	0.746	0.08 (0.14)	0.083
**Conscientiousness**								
Exp. Cons.	−0.08 (0.07)	0.309	−0.07 (0.07)	0.265	−0.15 (0.07)	0.043	−0.01 (0.11)	0.905
REG T2	−0.13 (0.07)	0.078	0.51 (0.06)	<0.001	−0.16 (0.07)	0.028	0.10 (0.05)	0.072
[Exp. Cons]^2^	−0.00 (0.08)	0.986	0.04 (0.06)	0.450	−0.01 (0.07)	0.342	−0.11 (0.10)	0.271
[REG T2]^2^	−0.07 (0.08)	0.332	−0.04 (0.06)	0.583	0.07 (0.07)	0.940	0.03 (0.06)	0.637
Exp.Cons. × REG T2	0.08 (0.06)	0.218	0.01 (0.07)	0.834	0.08 (0.08)	0.342	0.01 (0.07)	0.894
**Extraversion**								
Exp. Extra.	−0.02 (0.09)	0.858	−0.01 (0.08)	0.874	−0.03 (0.08)	0.721	0.11 (0.05)	0.024
SUR T2	−0.21 (0.08)	0.007	0.37 (0.07)	<0.001	0.07 (0.08)	0.377	−0.06 (0.10)	0.593
[Exp. Extra]^2^	−0.15 (0.09)	0.095	−0.03 (0.07)	0.635	−0.06 (0.07)	0.391	0.13 (0.04)	0.001
[SUR T2]^2^	−0.06 (0.08)	0.476	−0.02 (0.06)	0.709	0.06 (0.08)	0.407	−0.07 (0.09)	0.451
Exp.Extra. × SUR T2	−0.09 (0.09)	0.312	−0.10 (0.07)	0.197	0.02 (0.07)	0.773	0.08 (0.09)	0.340

NA, Negative Affectivity; REG, Regulation; SUR, Surgency; neuro, Neuroticism; cons, Conscientiousness; extra, Extraversion.

With regard to Regulation, we found that for mothers in both samples 1 and 2, higher infant Regulation was associated with more responsivity, with a quadratic effect significant for sample 1: the effect became stronger at higher levels of Regulation (see Table 5). For fathers in sample 1, and mothers in sample 2, higher Regulation was associated with more warmth. For mothers in sample 2, higher Regulation was also associated with less hostility.

For Surgency, we found that for mothers in sample 1, infant Surgency was associated with more warmth, whereas for fathers in sample 1 and mothers in sample 2, infant Surgency was associated with more responsiveness. For mothers in sample 2 infant Surgency was additionally associated with lower overprotection. Overall, the associations were all in the expected direction.

**Figure 3:**
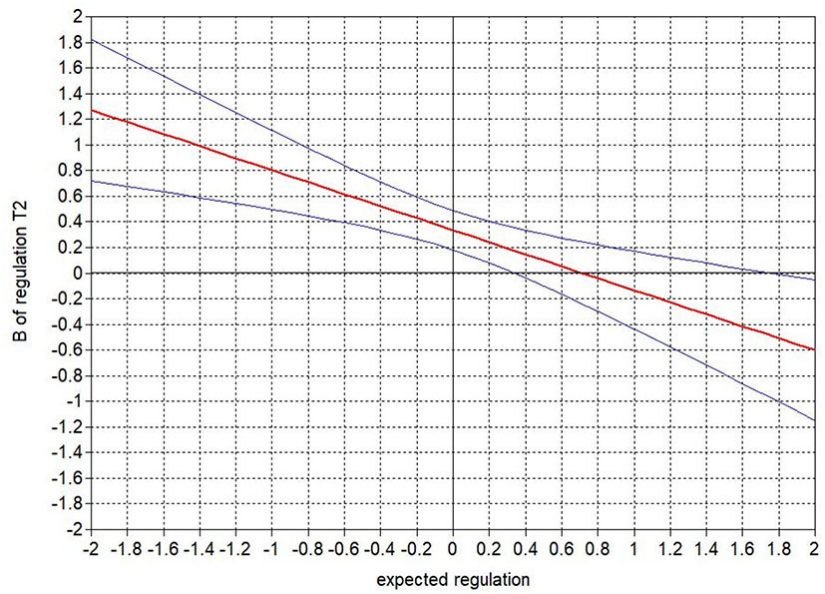
Johnson-Neyman interval for the association between Regulation at 4 months (mean centered) and maternal responsiveness, at the different levels of expected Regulation (mean centered).

Next, we examined the interactions between expected and newborn temperament in predicting parenting in the models (hypothesis 4). There was only one significant interaction: for mothers in sample 1, expected Regulation and actual Regulation interacted to predict responsiveness, such that for mothers who expected lower Regulation (effect significant up to 0.3 *SD* above the mean of expected Regulation), higher infant Regulation was related to more responsivity (or vice versa—lower Regulation was related to less responsivity). For mothers who expected levels of Regulation higher than 0.3 *SD* above the mean, infant Regulation was not related to their responsivity overall, except that for mothers who expected very high Regulation (effect significant from 1.8 *SD* above the mean), higher Regulation was related to less responsivity. For a graphical presentation of the Johnson-Neyman interval for the interaction effect, see Figure 3. None of the other interactions were significant.

To test the interactions, we also included the main effects of expected temperament on parenting behavior. Expected Negative Affectivity was associated with all parenting dimensions in sample 1: for mothers, higher expected infant Negative Affectivity was associated with lower warmth at 4 months, and for fathers with higher overprotection and hostility, and lower responsivity. There were no quadratic effects here. For sample 2, there were no significant effects for expected Neuroticism overall, but for its relation with responsivity, the quadratic effect was significant. When the quadratic effect is statistically significant, it should be interpreted together with the linear effect: higher levels of expected Neuroticism were associated with lower maternal responsivity, with the effect becoming stronger at higher levels of expected Neuroticism. With regards to expected regulation, for both mothers and fathers in sample 1, it was associated with more responsivity, and for mothers, it was also related to less overprotection. For mothers in sample 2, expected Conscientiousness was associated with lower hostility. Regarding expected Surgency, for mothers in sample 1, it was associated with more responsiveness and less overprotection, and the quadratic term for expected Surgency was significant for warmth, indicating that at lower and at higher levels of expected Surgency, warmth was lower. There was also a quadratic effect of expected Extraversion in sample 2, however, the main effect was significant here as well. Together, the effects in sample 2 indicated that higher expected Extraversion was associated with more warmth and that this effect became stronger at higher levels.

### Infant temperament at 12 months predicted by parenting

Our fifth hypothesis addressed relations between parenting at T2 and the development of infant temperament between T2 and T3. For mothers in sample 1, higher overprotection at 4 months predicted higher Negative Affectivity at 12 months, controlling for earlier Negative Affectivity (see Table 6). For mothers in sample 2, this association was not significant, whereas several other associations were: Regulation was predicted by higher levels of warmth, and Surgency was predicted by lower overprotection and hostility. For paternal parenting in sample 1, we found that higher hostility predicted more infant Negative Affectivity, whereas higher warmth predicted higher Surgency. Here we found one unexpected result: lower—rather than higher—paternal responsiveness was also associated with higher Surgency. Again, although most of the associations were in the expected direction, none of the specific links replicated across the two samples. For estimates, see Table 6.

**Table 6:** Effects of parenting and infant temperament at T2 on infant temperament at T3.

	**Mother**						**Father**					
	**NA T3**		**REG T3**		**SUR T3**		**NA T3**		**REG T3**		**SUR T3**	
**Predictors**	**β (S.E.)**	***p***	**β (S.E.)**	***p***	**β (S.E.)**	***p***	**β (S.E.)**	***p***	**β (S.E.)**	***p***	**β (S.E.)**	***p***
**T2**									
**Project 1**												
NA/REG/SUR T2	0.45 (0.07)	<0.001	0.46 (0.09)	<0.001	0.45 (0.08)	<0.001	0.42 (0.08)	<0.001	0.39 (0.09)	<0.001	0.49 (0.07)	<0.001
Overprotection	0.19 (0.09)	0.027	0.01 (0.09)	0.906	0.07 (0.09)	0.423	0.04 (0.08)	0.637	0.07 (0.10)	0.501	−0.02 (0.09)	0.819
Responsiveness	−0.17 (0.10)	0.089	0.11 (0.10)	0.258	−0.05 (0.10)	0.566	0.07 (0.11)	0.517	−0.01 (0.11)	0.931	−0.22 (0.09)	0.014
Hostility	–	–	–	–	–	–	0.31 (0.10)	0.001	0.01 (0.11)	0.941	−0.12 (0.09)	0.172
Warmth	0.10 (0.09)	0.270	0.03 (0.08)	0.682	0.09 (0.10)	0.369	0.11 (0.08)	0.185	0.17 (0.10)	0.101	0.21 (0.08)	0.014
**Project 2**												
NA/REG/SUR T2	0.76 (0.04)	<0.001	0.57 (0.08)	<0.001	0.42 (0.06)	<0.001						
Overprotection	0.07 (0.06)	0.228	0.05 (0.08)	0.570	−0.15 (0.07)	0.020						
Responsiveness	−0.05 (0.07)	0.485	−0.03 (0.10)	0.750	0.11 (0.08)	0.154						
Hostility T2	−0.12 (0.06)	0.050	0.06 (0.07)	0.393	−0.15 (0.07)	0.042						
Warmth	0.06 (0.07)	0.402	0.15 (0.06)	0.016	0.02 (0.06)	0.728						

NA, Negative Affectivity; REG, Regulation; SUR, Surgency.

Finally, to address hypothesis 6, we investigated whether continuity in temperament from T1 and T2 to T3 would be explained by the parenting variables at T2. We found no significant indirect effects either from expected temperament or newborn temperament, or their interaction, *via* parenting on 12-month temperament. For estimates of all indirect effects, see Table 7.

**Table 7:** Indirect effects of expected temperament, infant temperament T2, and their interaction, on temperament T3 *via* parenting T2.

	**Indirect effect**					
	**Project 1**				**Project 2**	
	**Mother**		**Father**		**Mother**	
**IV → DV**	**β (*S.E*.)**	***p***	**β (*S.E.)***	***p***	**β (*S.E*.)**	***p***
Exp. NA/neuro → NA T3	0.03 (0.03)	0.422	0.04 (0.04)	0.321	0.01 (0.02)	0.512
NA T2 → NA T3	0.06 (0.04)	0.088	0.06 (0.04)	0.134	−0.03 (0.03)	0.193
Exp. NA/neuro × NA T2 → NA T3	−0.02 (0.02)	0.482	0.04 (0.04)	0.278	0.01 (0.01)	0.684
Exp. REG/cons → REG T3	0.04 (0.04)	0.297	0.03 (0.02)	0.202	−0.01 (0.02)	0.592
REG T2 → REG T3	0.03 (0.03)	0.298	0.02 (0.03)	0.519	−0.02 (0.05)	0.734
Exp. REG/cons × REG T2 → REG T3	−0.03 (0.03)	0.321	−0.02 (0.03)	0.537	0.01 (0.01)	0.506
Exp. SUR/extra → SUR T3	−0.03 (0.03)	0.369	0.04 (0.03)	0.197	0.01 (0.02)	0.716
SUR T2 → SUR T3	0.01 (0.03)	0.690	−0.05 (0.04)	0.253	0.06 (0.04)	0.077
Exp. SUR/extra × SUR T2 → SUR T3	0.01 (0.01)	0.463	−0.00 (0.03)	0.914	0.00 (0.02)	0.918

IV, Independent Variable; DV, Dependent Variable; NA, Negative Affectivity; REG, Regulation; SUR, Surgency; neuro, Neuroticism; cons, Conscientiousness; extra, Extraversion.

## Discussion

The overall aim of this study was to examine how prenatal expectations of child characteristics provide a context for the early establishment of transactional associations between temperament and parenting. We found that parents had relatively optimistic expectations overall, that temperament expectations were positively associated with newborn temperament, and that perceived infant temperament predicted parenting behavior—with more difficult temperament (higher Negative Affectivity, lower Surgency, and Regulation) predicting less positive (lower responsiveness and warmth) and more negative (higher hostility and overprotection) parenting. While expected temperament and infant temperament were approximately equally strong predictors of parenting, there was little evidence for interactions between them, indicating either positive or negative surprises, in predicting parenting behavior. Parenting behavior did in turn predict temperament at 12 months, controlling for earlier infant temperament. Again, almost all associations were in the expected direction, with more negative and less positive parenting predicting a more difficult temperament. Parenting did not mediate associations between earlier and later temperament.

### Prenatal expectations of temperament

Our first hypothesis—that parents would expect their future child to have relatively favorable characteristics—was confirmed for both mothers and fathers, and in both samples. Similar to what people would indicate to be the ideal personality, parents expected infant temperament levels toward the ends of the scale but not at the extremes (Borkenau et al., 2009). Our findings are in line with findings that show that expecting parents have optimistic expectations overall (Harwood et al., 2007). At the same time, mean levels of infant Negative Affectivity and Regulation at 4–6 months were even more positive than expected. Previous studies also found that parents' reports of their infant's temperament at 3–4 months were more positive (i.e., less difficult, unadaptable, dull, and unpredictable) than they had expected during pregnancy (Mebert and Kalinowski, 1986; Diener et al., 2014). And similarly, general optimistic prenatal expectations about life after child birth have also been found to be exceeded half a year after childbirth (Harwood et al., 2007). This effect may be temporary; as a study that examined whether expectations were met at 12 months post-partum found that experiences were more negative than expected (Kalmuss et al., 1992). In the latter study, mean levels of the temperament traits had come closer to the prenatal expectations by 12 months than they were at 6 months. We add to previous findings on expectations about temperament by our inclusion of Surgency, for which infant ratings were actually more negative (i.e., lower) than parents expected. This may be important to investigate further as Western mothers of young infants have indicated Extraversion—which is conceptually related to Surgency—as the trait they would most like their children to score high on—more important than high Conscientiousness, low Neuroticism, or even high IQ (Latham and von Stumm, 2017).

Our second hypothesis was also confirmed overall, with expected temperament positively associated with infant temperament assessed at 4–6 months. The specific traits that were significant differed between mothers and fathers, and between the mothers across the two samples. A previous study also found different associations for mothers and fathers of the same children, with mothers' expectations associated with their ratings of the child's unpredictability and fussiness, whereas fathers' expectations were associated with their ratings of the child's dullness and unadaptability (Diener et al., 2014). Thus, our study found evidence of continuity in parents' expectations and their later perceptions of their child's temperament. This may reflect informant bias and/or actual parental ability to predict child temperament (e.g., based on parents' own temperament). The results of sample 1, where fathers' and mothers' ratings of newborn temperament were averaged, reducing informant bias, suggest at least some correspondence of expectations with actual temperament.

### Child temperament and parenting behavior

As prenatal expectations were positively associated with infant temperament, associations between infant temperament and early caregiving may be partially explained by these expectations. For each of the parenting behaviors, we found several associations with both parental expectations of temperament and infants' perceived temperament, which partly confirmed our third hypothesis. However, few of the specific links replicated across multiple samples—with the exception of expected Regulation and infant Regulation at 4–6 months with responsiveness, and infant Surgency with responsiveness. Additionally, some of the associations that differed between mothers and fathers in sample 1, which we might have interpreted as representing differences between maternal and paternal parenting, were similar for fathers of sample 1 and mothers of sample 2, making it unlikely that these differences represent differences between mothers and fathers more generally. A previous study found that early childhood effortful control was associated with more positive parenting for mothers and less negative control for fathers (Tiberio et al., 2016), whereas we found that Regulation—the related trait in infancy—was associated with more positive parenting for both mothers and fathers in sample 1, and to less hostility only for mothers in sample 2. In light of the lack of replication, we found across our samples between specific temperament and parenting dimensions, we will not discuss the results at the level of specific trait-parenting links or differences between mothers and fathers, but rather discuss more general trends.

A consistent finding was that all associations were in the direction that we had expected, with higher Negative Affectivity and lower Regulation and Surgency associated with more overprotection, hostility, and less responsiveness and warmth. Interestingly, there were only two associations between newborn Negative affectivity and parenting behavior, both in sample 2, whereas we did find several (i.e., 3) associations for *expected* Negative Affectivity. When infant Negative affectivity is assessed with parent report, pre-formed expectations may thus explain an important part of the effects of infant Negative affectivity. Overall, expected temperament was at least as much related to parenting at 4–6 months (12 significant associations) as newborn temperament was (10 significant associations), with similar effect sizes. So what appears to be a child effect, may actually oftentimes be a parent effect, with a negative view about what the unborn child's temperament will be like carrying over into the post-partum reality to predict caregiving. Similarly, a previous study found that prenatal optimism about life post-partum was more predictive of mother–infant bonding than whether or not expectations were confirmed (Robakis et al., 2015). Relatedly, a study found that prenatal expectations about how mothers would parent predicted children's distress to limitations (Perry et al., 2018). This raises questions of how specific the associations with parenting in our study are for prenatal views of the child, or whether these are already related then to expectations about parenting.

Comparing the different parenting dimensions, there were more significant associations of child temperament with responsiveness (10) than with the other parenting dimensions (4 for each). Responsiveness captures how parents feel they can respond effectively to the baby and is closely related to sensitivity, a key parenting dimension in attachment research (De Wolff and Van Ijzendoorn, 1997). Being about attunement to the child, responsivity may be associated more with the child's behavior and characteristics than the other parenting dimensions. In turn, these other parenting behaviors may be linked more strongly with parents' own emotions or traits, with parental anxiety linked to overprotection, positive affect to warmth, and anger to hostility. Future research could investigate whether different parenting behaviors vary in how much they are determined by the child's reactivity and regulation of emotions vs. the parents' own capacity to regulate their emotions.

In addition to the main effects of expected temperament and infant temperament, we expected in hypothesis 4 that expected temperament might determine the effect of infant temperament on parenting. However, we found only one such effect: In sample 1, infant Regulation was not predictive of maternal responsiveness when mothers expected high Regulation, whereas it was predictive for mothers who expected low Regulation. This effect might be an indication of the advantages of not having too positive expectations (Harwood et al., 2007), because at lower levels of expected Regulation, a high level of actual Regulation, a positive surprise, resulted in higher levels of responsiveness than at higher levels of expected Regulation, or a confirmed positive expectation. However, expecting low Regulation was also risky, because when mothers expected low Regulation and also reported low Regulation in their newborn, a confirmed negative expectation, mothers reported the lowest levels of responsiveness. It is important to note that only one out of all the interactions that were tested was positive, and this interaction did not replicate across samples, indicating that overall the combination of expected and infant temperament was less important than the independent effects of both expected temperament and infant temperament were. Given the knowledge that many factors affect parents' parenting above infant temperament (Taraban and Shaw, 2018), future studies may include parental factors such as parenting stress, coparenting, or parents' own temperament in addition to (expected) child temperament.

### Parenting behavior predicting infant temperament at 12 months

In line with our fifth hypothesis, parenting behavior at 4–6 months predicted infant temperament at 12 months, controlling for earlier levels of temperament at 4–6 months, with all temperament traits moderately stable. Again, almost all associations were in the direction of more negative and less positive parenting associated with more difficult temperament. We found only one association that was in the opposite direction: more paternal responsiveness at 4 months predicted lower Surgency at 12 months in sample 1. However, similar to the associations between temperament at 4–6 months and parenting, none of the specific links were replicated here across the two samples or parent genders. Past research has examined many specific parenting behaviors, such as sensitivity, mutual responsive orientation, limit setting, rejection, etc., as well as many different conceptualizations of temperament traits, such as fussiness, difficulty, attention, self-regulation, effortful control, irritability, etc. (Kiff et al., 2011). Results of this study show that it is important to be careful in interpreting all these links as differential effects, as they might not be as replicable as the more general trends with regard to negative parenting behaviors to levels of temperament traits that would signify maladjustment and positive parenting behaviors to levels of temperament traits that would signify better adjustment (Rothbart and Bates, 1998).

Our sixth and final hypothesis regarding the mediation of the association between earlier and later temperament by the parenting behaviors was not confirmed. We did not find any evidence of mediation effects. Temperament was quite stable, as can be expected, and was not very strongly and consistently influenced by parenting at this young age. A study examining mediation of earlier effortful control to later effortful control by maternal and paternal parenting found no significant effects at the youngest ages in their study (between 3 and 7 or between 5 and 11.5 years), and only one effect for maternal parenting at the oldest age, from 7 to 13.5 years (Tiberio et al., 2016). These mediational associations are then likely very small effects that perhaps slowly accumulate over time, only becoming visible later on in development, across larger age ranges.

### Strengths and limitations

This study has several strengths. First, we included two independent samples including longitudinal data for three waves. Second, in sample 1, the multi-informant approach allowed us to reduce mono-informant bias in the infant temperament measures. Third, previous studies have more often examined either multiple temperament traits or multiple parenting behaviors, but only very few longitudinal studies have included multiple traits—multiple parenting behavior associations (see Kochanska et al., 2004 for a notable exception).

In addition to these strengths, several limitations are also worth mentioning. First, both studies had a relatively small sample size, due to attrition across the three waves of the longitudinal study. This may have limited our power to detect the interaction and mediation effects. Second, although we did have data for fathers in sample 1, fathers were not included in sample 2. Therefore, we do not know whether the results from the fathers would replicate across samples. Future research is necessary to investigate whether associations between fathers and mothers differ. Third, although we could combine maternal and paternal reports of infant temperament for sample 1, thereby reducing mono-informant bias, this was not possible for sample 2. Including independent observations of infant temperament will be helpful in further elucidating how much of the association between expected and perceived temperament is due to the fact that parents may be correct in their predictions and base it on their knowledge of their own and their partner's characteristics that are genetically passed on to the child, and how much is due to bias of the parent in how they perceive their child (Stifter et al., 2008). Fourth, it is important to keep in mind that due to the lack of diversity in these samples, results may not generalize to samples of parents with fewer resources or different cultural backgrounds. Especially the expected characteristics may be dependent on cultural background, as well as how parents respond to these, as the desirability of different temperament traits is culturally determined (Desmarais et al., 2021).

## Conclusion

Prenatal expectations may provide a context that determines how parents view and respond to their child. Both prenatal expectations about children's temperament and perceptions of infant's temperament were equally and uniquely predictive of parents' early caregiving behavior. Early caregiving behavior in turn predicted changes in infants' temperament across the second half of their first year of life. Our findings may provide an avenue for preventive work with regard to early parenting problems; as prenatal expectations are associated with early caregiving, they may be helpful in identifying parents who are at risk of early maladaptive parenting behavior, with modifying these perceptions perhaps helpful. Especially parents' views of their future child as relatively difficult may signify potential problems, although for Surgency both very low and very high levels were associated with less warmth. It will be important to perform more studies of specific temperament-parenting links before any advice regarding specific parenting practices depending on children's temperament traits is formulated.

## Data availability statement

The raw data supporting the conclusions of this article will be made available by the authors, without undue reservation.

## Ethics statement

The studies involving human participants were reviewed and approved by Ethical Review Board Department Child Development and Education, University of Amsterdam. The patients/participants provided their written informed consent to participate in this study.

## Author contributions

AV contributed the analysis and interpretation of data for the work and drafted the work. All authors made substantial contributions to the conception or design of the work, acquisition of data, were involved in revising it critically for important intellectual content, provide approval for publication of the content, and agree to be accountable for all aspects of the work in ensuring that questions related to the accuracy or integrity of any part of the work are appropriately investigated and resolved.

## Conflict of interest

The authors declare that the research was conducted in the absence of any commercial or financial relationships that could be construed as a potential conflict of interest.

## Publisher's note

All claims expressed in this article are solely those of the authors and do not necessarily represent those of their affiliated organizations, or those of the publisher, the editors and the reviewers. Any product that may be evaluated in this article, or claim that may be made by its manufacturer, is not guaranteed or endorsed by the publisher.
